# Effect of Sr Deficiency on Electrical Conductivity of Yb-Doped Strontium Zirconate

**DOI:** 10.3390/ma15124126

**Published:** 2022-06-10

**Authors:** Adelya Khaliullina, Anastasia Meshcherskikh, Aleksander Pankratov, Liliya Dunyushkina

**Affiliations:** Institute of High Temperature Electrochemistry, 20 Akademicheskaya St., 620990 Ekaterinburg, Russia; adelia01@mail.ru (A.K.); lazyty@mail.ru (A.M.); a.pankratov@ihte.uran.ru (A.P.)

**Keywords:** Yb-doped strontium zirconate, strontium stoichiometry, conductivity, proton conduction

## Abstract

The effect of Sr-deficiency on microstructure, phase composition and electrical conductivity of Sr_x_Zr_0.95_Yb_0.05_O_3-δ_ (x = 0.94–1.00) was investigated via X-ray diffraction, scanning electron microscopy, energy-dispersive X-ray spectroscopy and impedance spectroscopy. The samples were synthesized by a chemical solution method and sintered at 1600 °C. According to X-ray diffraction data, the samples with x = 0.96–1.00 were single-phase oxides possessing an orthorhombic perovskite-type structure; while zirconia-based minor phases arouse at x = 0.94, which was confirmed by the electron microscopy. Sr stoichiometry was shown to influence the electrical conductivity. The highest total and bulk conductivities, 6–10^−4^ Scm^−1^ and 3–10^−3^ Scm^−1^, respectively, at 600 °C in humid air (pH_2_O = 3.2 kPa), were observed for the x = 0.98 composition. In the temperature range of 300–600 °C, the conductivity of the samples with x = 0.96–1.00 increased with the increase in humidity, which indicates a significant contribution of protonic defects to the charge transport. Electrical conductivity of Sr_x_Zr_0.95_Yb_0.05_O_3-δ_ was discussed in terms of the defect formation model and the secondary phases precipitation.

## 1. Introduction

Ceramic oxides exhibiting high oxide-ion and proton conductivity at the intermediate temperatures are of considerable interest for application as solid electrolytes in clean energy technologies. Proton-conducting solid oxide fuel cells (PC-SOFCs) have great potential for clean energy production by conversion of fuel chemical energy into electricity. Water splitting by using solid oxide electrolysis cells (SOECs) is a promising technology for the production of clean hydrogen which is currently considered as the fuel of the future. Proton-conducting SOFCs and SOECs operate at lower temperatures compared to the traditional oxide-ion conducting cells due to the high proton mobility. Proton conducting membranes are required to possess excellent ionic conductivity, as low as possible electronic conductivity, and chemical stability in oxidizing and reducing conditions, in order to provide high performance of the energy/fuel conversion and storage systems.

Alkaline-earth cerates and zirconates with a perovskite structure have been found to exhibit appreciable proton conduction in humid atmospheres in the intermediate temperature range (300–700 °C) [1,2,3,4,5,6,7,8,9]. Acceptor-doped barium and strontium cerates possess the highest proton conductivity among ABO_3_ perovskites; however, their poor chemical stability in atmospheres containing carbon and sulfur oxides, which is manifested in the formation of barium carbonate (sulfate) and CeO_2_-based oxides, hinders their practical use [1,7,10,11]. Thus, zirconates of strontium and barium, exhibiting lower proton conductivity, but higher chemical stability compared to the cerates, are considered as alternative candidates for the proton-conducting membranes of the solid oxide cells.

The study of the processes of hydration of the alkaline-earth zirconates [12,13], hydrogen isotope distribution, hydrogen solubility and diffusivity [14,15], the isotope effect on the electrical conductivity [16], and the effect of the water vapor partial pressure on the conductivity [4,5,6,17,18,19] regardless of certain differences, proved the proton incorporation and participation in the charge transport process in the perovskites. As demonstrated by multiple studies, the ionic (O^2−^ and H^+^) conductivity of the zirconates can be improved by substitution of zirconium with trivalent cations that promote the generation of oxygen vacancies to maintain charge balance, see e.g., [4,5,6,7,8,18,19].

Along with the acceptor doping, A-site stoichiometry was shown to influence the conducting behavior of ABO_3_ perovskites (A = Sr, Ba; B = Zr, Ce) [11,17,19,20,21]. Recently, we have reported that Sr_0.98_Zr_0.95_Y_0.05_O_3-δ_ composition exhibited the highest ionic conductivity among Sr_x_Zr_0.95_Y_0.05_O_3-δ_ (x = 0.94–1.02) series [19], which was interpreted as follows. A-site deficiency promotes the generation of additional oxygen vacancies that result in a rise of ionic conductivity; but on the other hand, it boosts the placement of the dopant ions into A-sites instead of B-sites. In A-sites, the trivalent cation acts as a donor impurity, which leads to a decrease in the oxygen vacancy concentration. As a result of these two opposite processes, the dependence of conductivity on Sr content has a maximum point at x = 0.98.

Yb-doped SrZrO_3_ was reported to possess a higher electrical conductivity compared to the Y-, Sc-, Er-, In-, Al- and Ga-doped strontium zirconates [5,6], which makes it more attractive for practical use. The solubility limit of ytterbium in SrZr_1–x_Yb_x_O_3-δ_ was reported to be about 5 at. %, and the conductivity was shown to increase with an increase in Yb concentration, reaching a maximal value for the SrZr_0.95_Yb_0.05_O_3-δ_ composition [22]. However, to the best of our knowledge, the influence of Sr content on the conducting properties of this perovskite have not yet been studied. Therefore, the aim of this work was to investigate the effect of Sr deficiency on the conductivity of Sr_x_Zr_0.95_Yb_0.05_O_3-δ_ (x = 0.94–1.00) electrolytes.

## 2. Materials and Methods

Sr_x_Zr_0.95_Yb_0.05_O_3-δ_ (x = 0.94, 0.96, 0.98, 1.00) powders were synthesized using ZrO(NO_3_)_2_·2H_2_O, Yb(NO_3_)_3_·nH_2_O and SrCO_3_ (all with 99% purity) as precursors via a chemical solution method. Solutions of zirconyl nitrate dehydrate in distilled water and ytterbium nitrate hydrate in ethanol, with concentrations of 62.9 g of ZrO_2_ and 24.4 g of Yb_2_O_3_ per 1 L, respectively, were made. The solutions were mixed with SrCO_3_ powder in the ratios corresponding to the nominal compositions. Then, citric acid and glycine were added (the molar ratio of metal cations, glycine, and citric acid was 2:2:1). The solution was stirred and heated on a hot plate to evaporate the solvent until its ignition occurred, followed by calcining at a temperature of 1200 °C for 2 h. The obtained substance was reground into powder and pressed at 130 MPa. The pellets were then sintered in air at 1600 °C for 5 h.

The relative density of the sintered samples was determined by the Archimedes method using kerosene as a liquid. Crystal structure and phase composition of the obtained materials were investigated with the X-ray diffraction (XRD) method with Cu Kα radiation (D-Max 2200, Rigaku, Tokyo, Japan). Diffraction was carried out in the range from 10° to 90° at a scanning speed of 0.02°/min and step of 0.1°.

For the microstructure and composition study, the sintered pellets were polished, finishing with 1 μm grit diamond paste, and were thermally etched at a temperature of 1400 °C for 4 h to make the grain boundaries visible. The surface of the samples was studied using a MIRA 3 LMU (Tescan, Brno, Czech Republic) scanning electron microscope (SEM) equipped with an energy-dispersive X-ray spectroscopy (EDX) system (Oxford Instruments X-MAX 80, Abingdon, UK). For the chemical composition study, EDX data were collected and averaged for 10 spots.

Electrical conductivity of the samples was studied using two-probe AC impedance spectroscopy (Parstat 2273-SVS, Advanced Measurement Technology Inc., Oak Ridge, TN, USA) in a frequency range of 0.1 Hz–1 MHz with an amplitude of 30 mV. For the impedance measurements, platinum electrodes were applied to the pellets by painting Pt paste and were sintered at 1000 °C for 1 h in air. The surface area of the electrodes was 0.5 cm^2^, the thickness of the pellets was 0.2 cm. The impedance measurements were carried out in the temperature range of 300–800 °C in air with controlled humidity. The water vapor partial pressure, pH_2_O, was held constant by passing the gas flow through a column filled with zeolites (pH_2_O = 0.04 kPa) or through a water bubbler kept at 0 °C and 25 °C (pH_2_O = 0.61 kPa and 3.2 kPa). Zview 2 software was used for the fittings of the measured Nyquist plots.

## 3. Results

### 3.1. Microstructure, Phase and Chemical Composition of Sr_x_Zr_0.95_Yb_0.05_O_3-δ_ Samples

**Figure 1:**
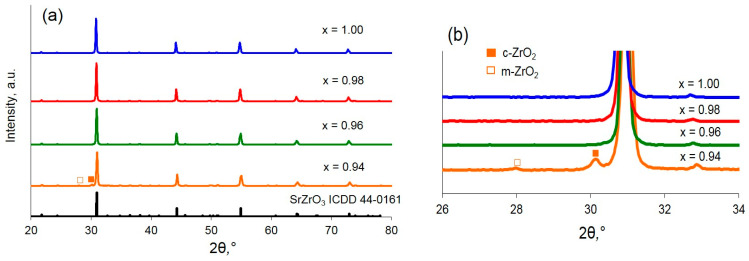
XRD patterns of the samples Sr_x_Zr_0.95_Yb_0.05_O_3-δ_: (**a**) in the range of 20–80°, (**b**) enlarged XRD pattern between 26° and 34°.

XRD patterns of the obtained Sr_x_Zr_0.95_Yb_0.05_O_3-δ_ samples presented in Figure 1 were indexed on the basis of the SrZrO_3_ orthorhombic structure as confirmed by the corresponding ICDD file (44–0161). In the case of x ≥ 0.96, no traces of minor phases were observed. For x = 0.94, except for the peaks of the major orthorhombic phase, two minor reflexes at 28° and 30.1° were recorded as can be clearly seen in Figure 1b. These reflexes were ascribed to ZrO_2_ monoclinic structure and Yb-stabilized ZrO_2_ cubic structure. Based on the XRD data, it can be concluded that the Sr-deficient homogeneity boundary in Sr_x_Zr_0.95_Yb_0.05_O_3-δ_ lays between x = 0.94 and x = 0.96; at the lower Sr contents, ZrO_2_-based minor phases arise.

**Figure 2:**
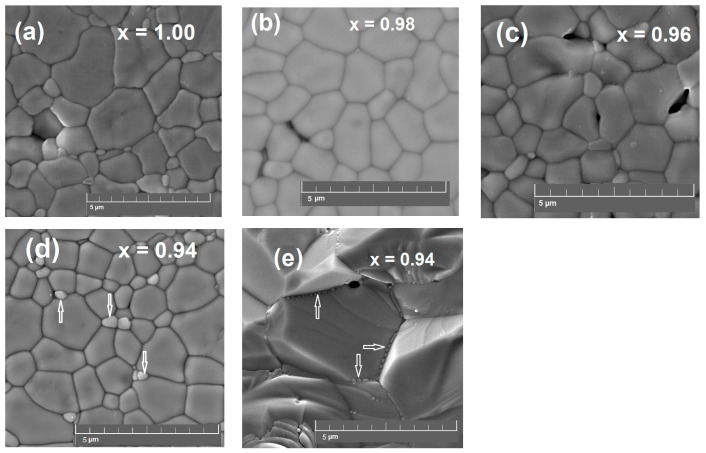
SEM images of Sr_x_Zr_0.95_Yb_0.05_O_3-δ_: the surface of (**a**) x = 1.00, (**b**) 0.98, (**c**) 0.96 and (**d**) 0.94 samples after polishing and thermal etching, (**e**) fracture of the x = 0.94 sample. The impurity phase grains are marked with arrows.

The relative density of the sintered samples varied in the range of 90–95% with respect to the theoretical density. SEM images of the surface of Sr_x_Zr_0.95_Yb_0.05_O_3-δ_ samples shown in Figure 2a–c reveal dense microstructure with grains reaching 2.5 μm in size. For the sample with the strongest Sr deficiency (x = 0.94), the light grey grains with a diameter of 0.3–0.5 μm embedded in the large grain matrix are observed. Figure 2d displays the fracture surface of the x = 0.94 sample; the minor phase nanograins precipitated at the intergrain boundary can be clearly seen.

According to the EDX mapping images shown in Figure S1 and the measured element concentrations summarized in Table S1, the light grey grains observed in the SEM-image of the sample with x = 0.94 contain mostly zirconium and ytterbium in the ratio close to 6:5. Taking into account the appearance of the X-ray reflection associated with the cubic ZrO_2_-based phase, these grains can be considered as the precipitated phase of Yb-doped ZrO_2_.

So, the obtained XRD, SEM and EDX data indicate that a decrease in the atomic concentration of strontium below 0.96 leads to nucleation of ZrO_2_-based phases. Recently, the similar regularity was observed for the undoped and Y-doped SrZrO_3_ [19]. The process of the secondary phases’ precipitation might be enhanced by the evaporation of strontium during the sintering of the ceramics. The surface alkaline earth metal depletion has been previously reported for the doped SrZrO_3_ and BaZrO_3_ ceramics [17,19,21,23] and explained by evaporation of the alkaline earth metals during the high-temperature treatment of ceramics.

### 3.2. Electrical Conductivity of Sr_x_Zr_0.95_Yb_0.05_O_3-δ_

**Figure 3:**
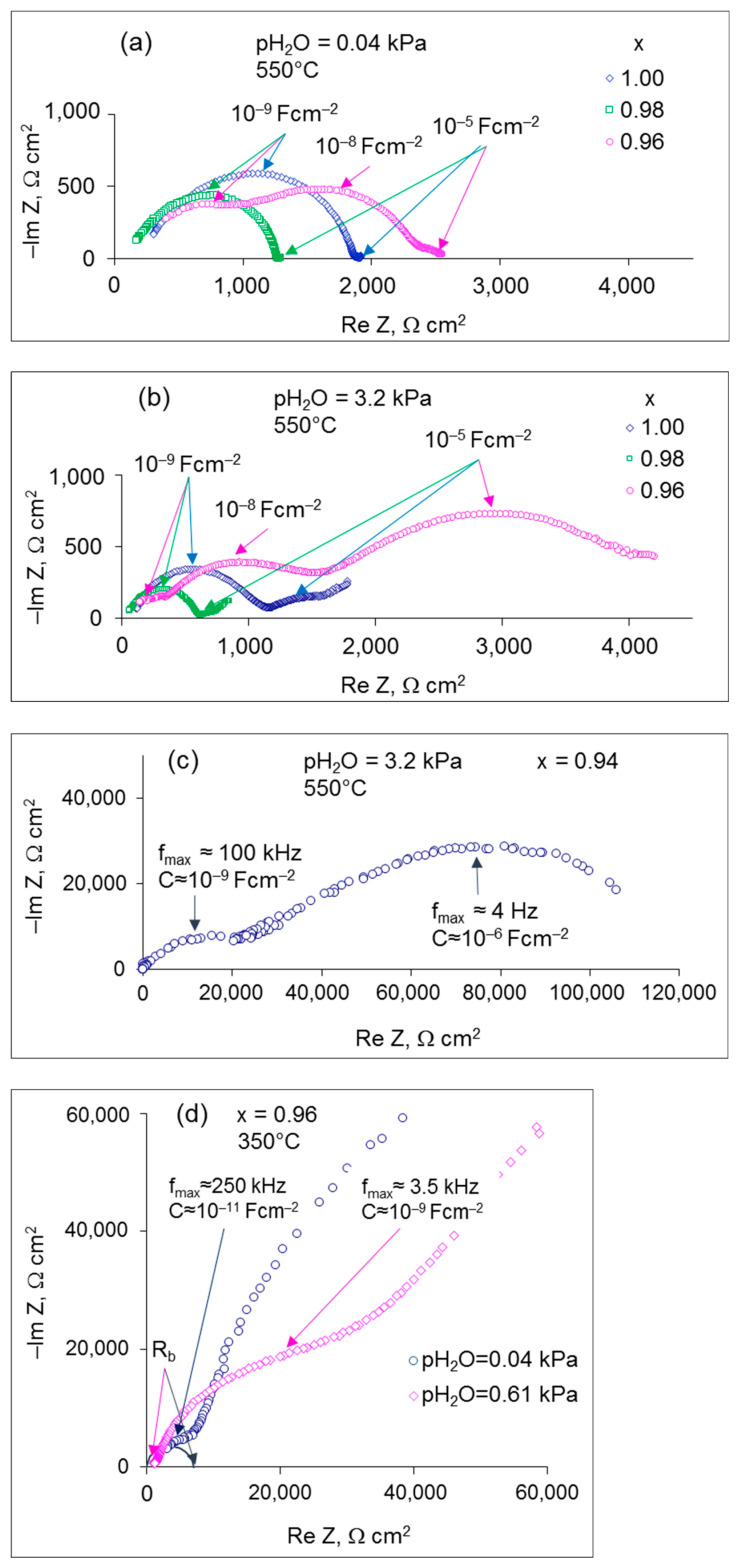
The impedance spectra for x = 0.96, 0.98 and 1.00 samples measured at 550 °C under (**a**) pH_2_O = 0.04 kPa and (**b**) 3.2 kPa; (**c**) x = 0.94, pH_2_O = 3.2 kPa, 550 °C; (**d**) enlarged high-frequency fragments of the hodographs of the x = 0.96 sample at 350 °C.

The electrical conductivity of the investigated samples was studied in the air with controlled humidity (pH_2_O = 0.04, 0.61 and 3.2 kPa) over the temperature range of 300–800 °C. To illustrate the influence of Sr content on the conduction features of Sr_x_Zr_0.95_Yb_0.05_O_3-δ_, the impedance spectra of the samples with different Sr contents measured in dry (pH_2_O = 0.04 kPa) and wet (pH_2_O = 3.2 kPa) air at 550 °C are presented in Figure 3a,b. The hodographs measured at other temperatures and pH_2_O are given in Figure S3. As can be seen, Sr deficiency has a significant impact on the shape of the hodographs. At the lowest Sr content (x = 0.94), the total resistance increases by more than an order of magnitude, which is why the hodographs of this sample are shown in a separate plot (Figure 3c, S3m).

In dry air (pH_2_O = 0.04 kPa), the compositions with x = 0.98 and 1.00 exhibit a semicircle with the characteristic capacitance of ~5–10^−9^ F cm^−2^ at the characteristic frequency f_max_ (at maximum imaginary part of Z) of ~20 kHz and a small low-frequency arc (f_max_ ~5 Hz) with a capacitance of 10^−5^ F cm^−2^ (Figure 3a). These spectra were deconvoluted with the help of the equivalent circuit shown in Figure S2a. Based on the obtained capacitance values, the high-frequency semicircle was ascribed to the grain boundary response. The high-frequency intersection of the semicircle with the real impedance axis was assigned to the bulk resistance of the electrolyte. At temperatures below 550 °C, a high-frequency arc with the characteristic capacitance of ~10^−11^ F cm^−2^ starts to swell, which can be associated with the bulk response of the electrolyte; in these cases, the hodographs were described by the equivalent circuit presented in Figure S2b.

In contrast to x = 1.00 and 0.98 samples, the Nyquist plot of the x = 0.96 composition consists of three overlapping arcs at the characteristic frequencies of ~135 kHz, ~5 kHz and ~3 Hz with the corresponding capacitances of ~10^−9^, 10^−8^ and 10^−5^ F cm^−2^. At lower temperatures in dry air (350 °C, pH_2_O = 0.04 kPa), another high-frequency arc with the characteristic capacitance of ~10^−11^ F cm^−2^ appears, which is apparently caused by the bulk response of the electrolyte (see Figure 3d and the equivalent circuit in Figure S2c). The low-frequency arc capacitance value (~10^−5^ F cm^−2^) is typical for the charge transfer processes at the electrode/electrolyte interface; so, this arc was attributed to the interfacial contribution. The semicircles with the capacitances of ~10^−9^ and 10^−8^ F cm^−2^ can be associated with the grain boundary contribution and the precipitated minor phases response. As discussed above, the nominal Sr-deficiency and the evaporation losses initiate nucleation of ZrO_2_-based nanograins at the grain boundaries of the ceramic samples. We suppose that at x = 0.96, the minor phase contribution becomes visible on hodographs at medium frequency even though the impurities are not yet detectable by X-rays. The nanoscale size of the precipitated zirconia grains is responsible for the fact that their contribution is characterized by the capacitance of ~10^−8^ F cm^−2^, which is typical for grain boundaries.

The sample with x = 0.94 exhibits a significantly larger impedance compared to other compositions as can be seen in Figure 3c. Two arcs with the characteristic capacitance of ~10^−9^ F cm^−2^ and ~10^−6^ F cm^−2^ can be distinguished on the hodograph measured under pH_2_O = 3.2 kPa at 550 °C, which was deconvoluted with the help of the equivalent circuit shown in Figure S2d. The two arcs were assigned to the electrolyte response and the electrode polarization, respectively. A reliable deconvolution of the impedance spectra into the bulk and grain-boundary processes was not possible. Change of pH_2_O had a weak effect on the shape of the impedance spectra of the x = 0.94 sample.

**Figure 4:**
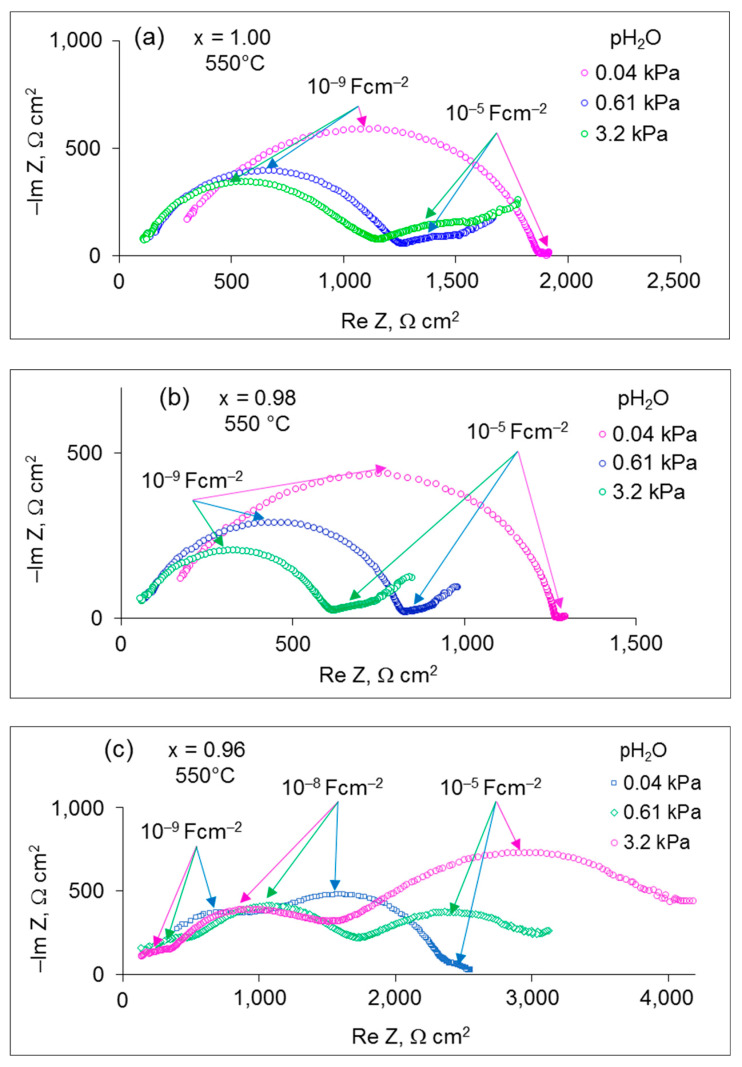
The impedance spectra for (**a**) x = 1.00, (**b**) x = 0.98 and (**c**) x = 0.96, measured under different pH_2_O at 550 °C.

To demonstrate the effect of humidity on the conducting properties of the x = 0.96–1.00 samples, the Nyquist plots of the samples measured at different pH_2_O values are depicted in Figure 4. With increasing pH_2_O, both the bulk and grain boundary responses of the samples with x = 0.96–1.00 shrink, which can be explained by the growing proton conduction; while the interfacial response with the characteristic capacitance of ~10^−5^ F cm^−2^ significantly increases. Besides, a lower-frequency tail emerges in the spectra, which can be caused by slow gas diffusion through the porous electrodes.

The total resistance R_t_ of the electrolyte is a sum of R_b_ and R_gb_ (R_b_ and R_gb_ denote the grain interior and grain boundary resistances). The grain interior, grain boundary and total conductivities were calculated using the obtained values of R_b_, R_gb_ and R_t_ by the following formula:

σ = L/SR,(1)

where L denotes the sample thickness, S is the electrode area, and R is the related resistance.

**Figure 5:**
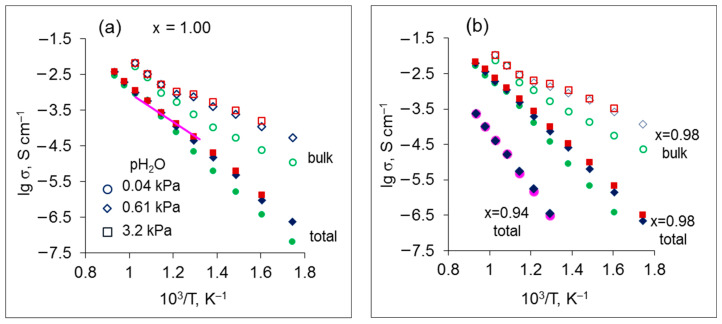
Arrhenius plots of the total and bulk conductivities of Sr_x_Zr_0.95_Yb_0.05_O_3-δ_ in air with different pH_2_O values: (**a**) x = 1.00, (**b**) x = 0.94 and 0.98. Solid and open symbols denote total and bulk conductivity, respectively; circles, diamonds and squares correspond to pH_2_O = 0.04 kPa, 0.61 kPa and 3.2 kPa, respectively. A solid line represents the total conductivity of SrZr_0.95_Yb_0.05_O_3-δ_ reported in [22].

Figure 5 displays the Arrhenius dependences of the total and bulk conductivities of Sr_x_Zr_0.95_Yb_0.05_O_3-δ_ samples. For all compositions, the conductivity of grain boundaries was close to the total conductivity, so it was not shown in order to avoid cluttering the figure. The total conductivities of all samples are linear in the Arrhenius plane in the whole investigated temperature range, while the grain interior conductivity dependences exhibit an inflection at ~600 °C. At temperatures above 600 °C, the slopes of the bulk and total conductivity are similar, which means that the corresponding activation energies possess close values. Below the inflection point, the slope of the bulk conductivity decreases. Further, in the high temperature region (above 600 °C), the conductivity of grain bulk does not change with humidity, while at lower temperatures, it increases with a rise of pH_2_O as can be seen in Figure 5. It is worth noting that the total conductivity of Sr_x_Zr_0.95_Yb_0.05_O_3-δ_ (x = 1.00) obtained in the present research is similar to that of the same composition measured in air and reported in [22]. The literature data are presented in Figure 5a for comparison.

The activation energies of the bulk and total conductivities of Sr_x_Zr_0.95_Yb_0.05_O_3-δ_ are summarized in Table 1. As can be seen, the activation energy of the bulk conductivity is sensitive to Sr deficiency and pH_2_O: it decreases with decreasing x from 1.00 to 0.96 and with a rise of pH_2_O. The effect of pH_2_O can be explained by hydration of the samples and the appearance of protonic defects in humid atmospheres at relatively low temperatures. Water molecules incorporate into the lattice of Sr_x_Zr_0.95_Yb_0.05_O_3-δ_, which results in the generation of protonic defects according to the following reaction:

(2) $$H_{2}O+V_{O}^{..}+O_{O}^{\times }\;↔2OH_{O}^{.},$$

where $O_{O}^{\times }$ is an oxygen ion in a normal lattice site, $V_{O}^{..}\;$ is an oxygen vacancy, $OH_{O}^{\cdot }$ denotes a proton localized on an oxygen ion, or a protonic defect.

At low temperatures, Reaction (2) proceeds to the right to produce the protonic defects in the oxides. According to the law of mass action, the concentration of the bulk protonic defects increases proportionally to pH_2_O^1/2^. As temperature increases, the dehydration process progresses, with release of the proton defects from the oxide. So, in the low temperature range (below 600 °C), the bulk conductivity of the samples with x = 0.96–1.00 is mainly protonic, while at higher temperatures the contribution of protons in the charge transfer process decreases. Change of the dominant charge carriers causes the change of the bulk conductivity slope observed in the Arrhenius plots at ~600 °C (see Figure 5).

The total conductivity behavior is restricted by grain boundaries; as a result, an increase in pH_2_O leads to a slight decrease in the activation energy, whereas Sr content does not influence the activation energy of the total conductivity at x = 0.96–1.00. As to the x = 0.94 sample, the activation energy of the total conductivity is much higher than for other compositions and does not vary with a change of pH_2_O, which indicates a negligibly small contribution of protons in the total conductivity.

**Figure 6:**
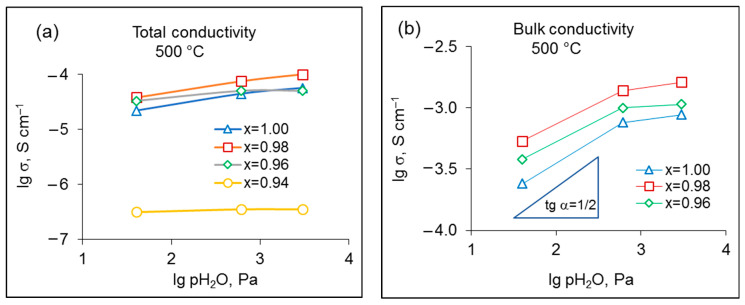
Total (**a**) and bulk (**b**) conductivity of Sr_x_Zr_0.95_Yb_0.05_O_3-δ_ samples as a function of pH_2_O at 500 °C. Lines between points are only a guide to the eye.

Figure 6 shows the total and bulk conductivities of Sr_x_Zr_0.95_Yb_0.05_O_3-δ_ samples as functions of pH_2_O at 500 °C in a logarithmic scale. The conductivity of the x = 0.96–1.00 samples increases with increasing pH_2_O, which indicates the participation of protons in the process of charge transport. As can be seen in Figure 6b, at low humidity values, the slope of the bulk conductivity is close to ½, being in agreement with Reaction (2); as the humidity increases, the slope decreases, which can be caused by saturation of the oxides with H_2_O.

Small deficiencies in Sr boost the conductivity of the strontium zirconate: the bulk conductivity of the x = 0.98 composition is about 2.2 times more than that of x = 1.00 over the studied pH_2_O range. This can be explained by the increased oxygen vacancy concentration to maintain the charge neutrality according to the following reaction:

(3) $$-SrO\;\to V_{Sr}^{\prime \prime }+V_{O}^{..},$$

where $V_{Sr}^{\prime \prime }$ is a vacancy of strontium.

The observed decline of the bulk conductivity upon further increase in Sr deficiency (x < 0.98) can be caused by partitioning of Yb^3+^ ions over A- and B-sites in the doped A^2+^B^4+^O_3_ perovskite. Substitution of Zr^4+^ with Yb^3+^ results in a rise of the oxygen vacancy concentration according to Reaction (4), and thus, in an increase in the ionic conductivity:

(4) $${\mathrm{Yb}}_{2}O_{3\;}\left(-{\mathrm{ZrO}}_{2}\right)\to 2Yb_{Zr}^{\prime }+3O_{O}^{\times }+V_{O}^{..}.$$

However, incorporation of Yb^3+^ into Sr^2+^-sites has the opposite effect on the oxygen vacancy concentration:

(5) $${\mathrm{Yb}}_{2}O_{3\;}\left(-\mathrm{SrO}\right)+V_{O}^{..}\to 2Yb_{Sr}^{\cdot }+3O_{O}^{\times }.$$

In Reactions (4) and (5), $Yb_{Zr}^{\prime }\;\mathrm{and}\;Yb_{Sr}^{\cdot }$ denote the substitution defects in zirconium and strontium sites.

One can expect that the decreasing Sr concentration in Sr_x_Zr_0.95_Yb_0.05_O_3-δ_ disposes the placement of the dopant ions in A-sites. This trend was confirmed for the Sr-deficient zirconates doped with yttrium by Raman spectroscopy [23]. In this case, an increase in the Sr deficiency leads to a decrease in the ionic conductivity.

The total conductivity of Sr_x_Zr_0.95_Yb_0.05_O_3-δ_ is less sensitive to the Sr concentration because of the masking effect of grain boundaries (see Figure 6a). A sharp decrease in the total conductivity at x = 0.94 can be explained by the nucleation and growth of the minor zirconia-based phases including the poorly conducting monoclinic zirconia, which hinders the transport of oxide ions and protonic defects.

The data on the influence of the Sr deficiency on the electrical conductivity of Yb-doped strontium zirconate obtained in the present research show the importance of controlling the concentration of the alkaline earth elements in zirconates. As was mentioned above, SrO evaporates during sintering of the ceramics, which can lead to deviations from the stoichiometry and an undesirable increase in the electrolyte resistance.

It is of importance to consider the influence of pH_2_O on the electrode behavior. As can be seen from the impedance spectra (Figure 3 and Figure 4), the interfacial polarization rises with increasing pH_2_O, which can be explained by suppressing the hole conductivity because of the incorporation of water molecules into the solid oxide lattice [24]. In dry oxidizing atmospheres, the interaction of the oxides with oxygen results in the generation of electron holes as described by the following reaction:

(6) $$\frac{1}{2}{\;O}_{2}+V_{O}^{..}↔O_{O}^{\times }+2h^{.}.$$

The presence of electron holes facilitates the charge transfer reactions at the electrode/electrolyte interface. In humid air, the incorporation of water (Reaction (2)) competes with that of oxygen (Reaction (6)), which leads to a decrease in the hole concentration; as a result, the interfacial polarization increases as pH_2_O rises. In the high temperature range, dehydration of the materials causes the hole concentration to increase, which alleviates the electrode polarization. Indeed, the polarization resistance of the studied cells was nearly independent of humidity at high temperatures (750–800 °C).

Recently, we reported the conductivity of the Sr_x_Zr_0.95_Y_0.05_O_3-δ_ series (x = 0.94–1.02) [19]. It is worth comparing the influence of the dopant type on the transport properties of the zirconates. In both yttrium and ytterbium doping, the Sr-deficient compositions (x = 0.98) exhibits the highest conductivity. The bulk conductivity of Sr_0.98_Zr_0.95_Yb_0.05_O_3-δ_ is about ten times higher than that of Sr_0.98_Zr_0.95_Y_0.05_O_3-δ_. This can be explained by a larger number of the electron shells in ytterbium and the greater shielding effect experienced by the ionic charge carriers ($V_{O}^{..}$ and $OH_{O}^{\cdot }$), which provides the higher charge carriers mobility. Therefore, doping with Yb is preferable to raising the conductivity.

## 4. Conclusions

The effect of Sr stoichiometry on microstructure, phase composition and electrical conductivity of Sr_x_Zr_0.95_Yb_0.05_O_3-δ_ (x = 0.94–1.00) was investigated via XRD, SEM, EDX and impedance spectroscopy. The samples were synthesized by the chemical solution method using ZrO(NO_3_)_2_·2H_2_O, Yb(NO_3_)_3_·nH_2_O and SrCO_3_ as precursors, and sintered at 1600 °C, 5 h. The x = 0.96–1.00 samples were shown to be single-phase; the further increase of Sr-deficiency promoted the nucleation process of zirconia-based phases.

Small Sr deficiency was shown to improve the conductivity due to the formation of additional oxygen vacancies; as a result, the x = 0.98 composition exhibited the highest total and bulk conductivity, which reached 6–10^−4^ Scm^−1^ and 3 ∙ 10^−3^ Scm^−1^ at 600 °C in wet air (pH_2_O = 3.2 kPa). A decrease in the conductivity at stronger Sr deficiencies can be explained by the partitioning of the dopant ions (Yb^3+^) over A- and B-sites in the doped A^2+^B^4+^O_3_ perovskite and the related decrease in the oxygen vacancy concentration. A sharp decrease in the total conductivity at x = 0.94 was explained by the nucleation of the minor zirconia-based phases including the poorly conducting monoclinic zirconia. The conductivity of the samples with x = 0.96–1.00 increased with the increase in humidity in the temperature range of 300–600 °C, which indicates a notable contribution of protons to the charge transport. The conductivity of Yb-doped strontium zirconate exceeds that of Y-doped zirconate, which makes it a more prospective material for application in PC-SOFCs.

## Figures and Tables

**Table 1:** Activation energies of the total and bulk conductivities of Sr_x_Zr_0.95_Yb_0.05_O_3-δ_.

	Ea, Tot, kJ mol^−1^ (300–800 °C)		Ea, Bulk, kJ mol^−1^ (300–600 °C)		
pH_2_O, kPa	x = 0.96–1.00	x = 0.94	x = 1.00	x = 0.98	x = 0.96
0.04	112 ± 5	145 ± 8	60 ± 2	58 ± 2	58 ± 2
0.61	98 ± 5		49 ± 2	43 ± 2	39 ± 2
3.2	95 ± 5		45 ± 2	39 ± 2	32 ± 2

## Data Availability

Not applicable.

## Supplementary Materials

The following supporting information can be downloaded at: https://www.mdpi.com/article/10.3390/ma15124126/s1, Figure S1: (a) Back scattered electron image of x = 0.94 sample and (b) corresponding EDX mapping images of Sr, Zr, Yb and O; Figure S2: Equivalent circuits used for deconvolution of impedance spectra for: (a) x = 0.98 and 1.00 at 550–800 °C; (b) x = 0.98 and 1.00 at 500 °C and below; (c) x = 0.96 at 350 °C and below; (d) x = 0.94. R_t_ denotes the total resistance of a sample; R_b_, R_gb_, R_mp_ and R_e_ are the grain interior, grain boundary, minor phase and electrode resistances, respectively; Q_b_, Q_gb_, Q_mp_ and Q_e_ are the constant phase element associated with the grain interior, grain boundary, minor phase and electrode responses; Q_t_ is the constant phase element related with the total re-sponse of a sample; Figure S3: Impedance spectra of the samples with x = 0.96–1.00 measured in air at 700 °C (a–c), 600 °C (d–f), 500 °C (g–i) and 400 °C (j,k); at pH2O = 0.04 kPa (a,d,g,j), 0.61 kPa (b,e,h,k) and 3.2 kPa (c,f,i); and the spectra of the x = 0.94 sample at pH2O = 0.04 kPa (l); Table S1: Elemental composition (at.%) of the surface of of x = 0.94 sample after polishing and ther-mal etching (1400 °C, 4 h) from EDX data.
