# Supplementary material for: Effect of Sr Deficiency on Electrical Conductivity of Yb-Doped Strontium Zirconate
Source: Materials (Basel). 2022 Jun 10;15(12):4126. doi: 10.3390/ma15124126 (PMC9229988; doi:10.3390/ma15124126)
Supplement: Supplementary file 1 [file materials-15-04126-s001.zip › materials-1747681-supplementary.pdf]

materials

Supplementary Data

Effect of Sr deficiency on electrical conductivity of Yb-doped strontium zirconate

Adelya Khaliullina[1], Anastasia Meshcherskikh[1], Aleksander Pankratov[1] and Liliya Dunyushkina [1,*]

[1] Institute of High Temperature Electrochemistry, 20 Akademicheskaya St, 620990 Ekaterinburg, Russia; adelia01@mail.ru (A.K.); lazyty@mail.ru (A.M.); a.pankratov@ihte.uran.ru (A.P.)
* Correspondence: lidung@list.ru; Tel.: +7(343)3623240

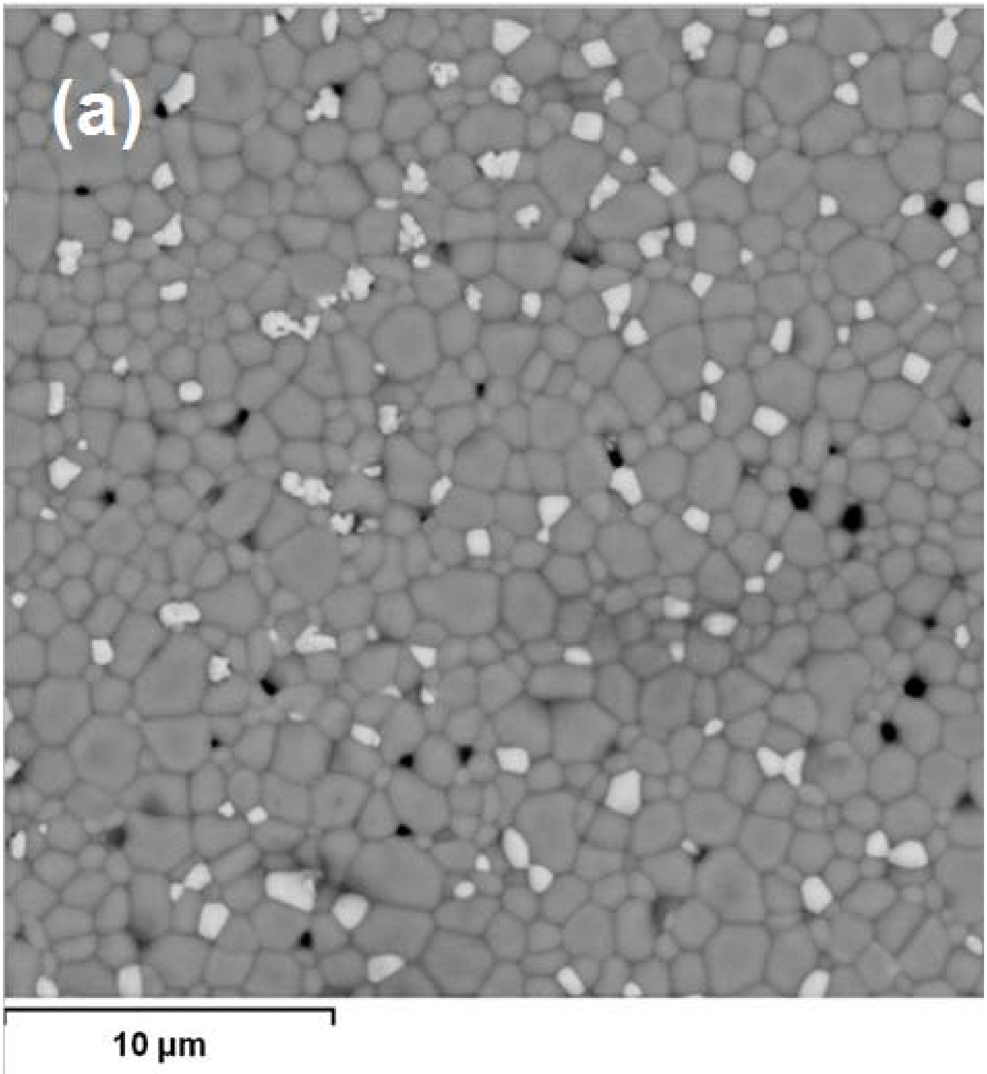

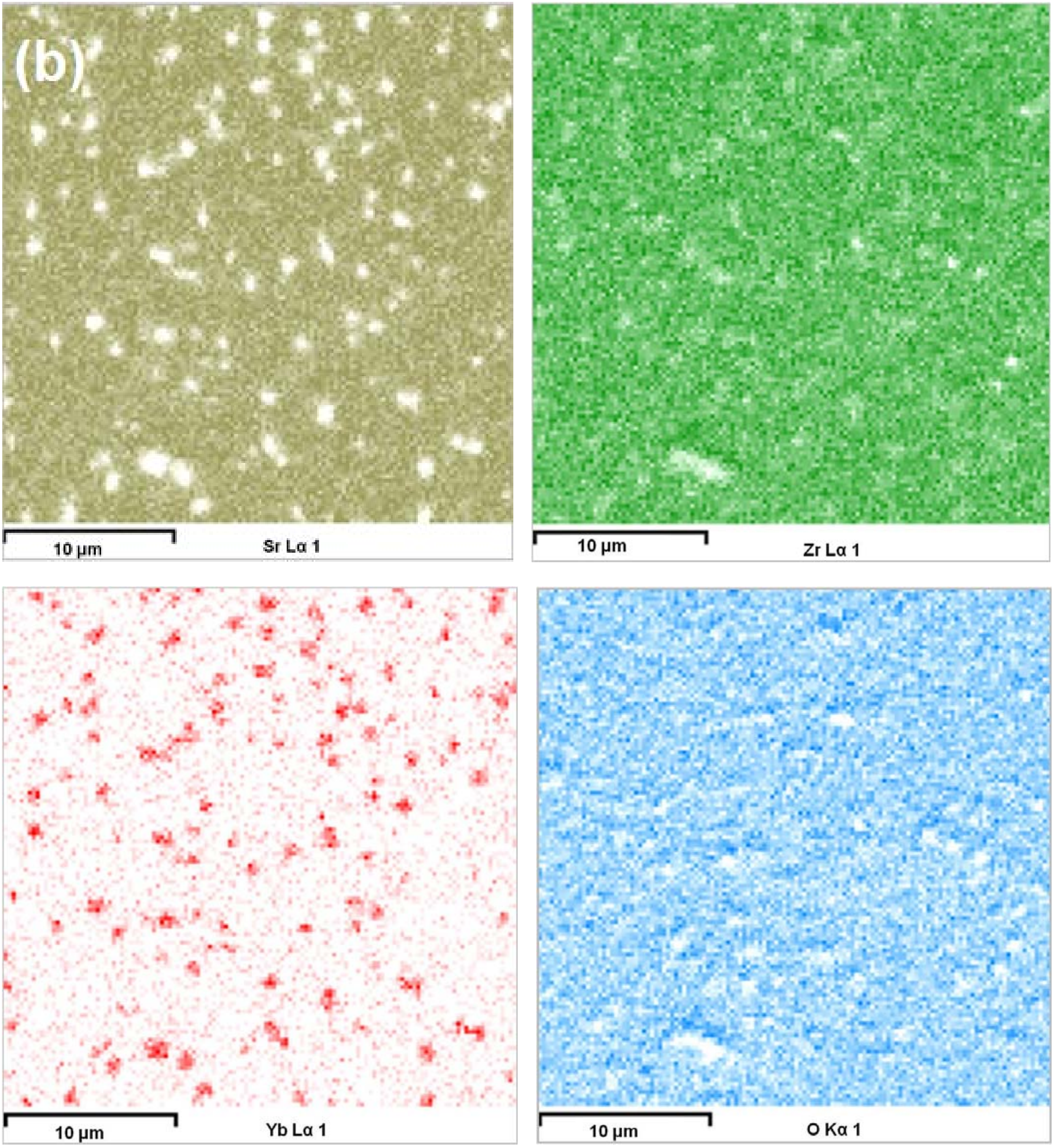

Figure S1. (a) Back scattered electron image of x = 0.94 sample and (b) corresponding EDX mapping images of Sr, Zr, Yb and O.

Table S1. Elemental composition (at.%) of the surface of of x = 0.94 sample after polishing and thermal etching (1400 °C, 4 h) from EDX data

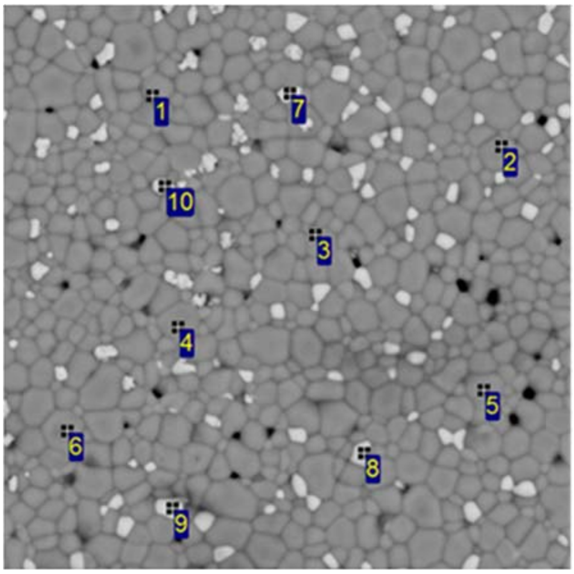

Point	O	Sr	Zr	Yb
1	61.83	18.54	18.86	0.77
2	57.66	20.70	20.86	0.78
3	57.90	20.49	20.73	0.88
4	56.58	20.93	21.14	0.72
5	57.21	20.71	21.05	0.81
6	56.62	21.21	21.39	0.78
Average values for points 1–6 (main phase)				
	**58.00**	**20.43**	**20.67**	**0.79**
7	59.57	8.94	18.09	13.39
8	62.21	4.68	17.56	15.54
9	57.23	4.33	20.14	18.30
10	58.67	7.78	18.38	15.16
Average values for points 7–10 (secondary phase)				
	**59.42**	**6.43**	**18.54**	**15.60**

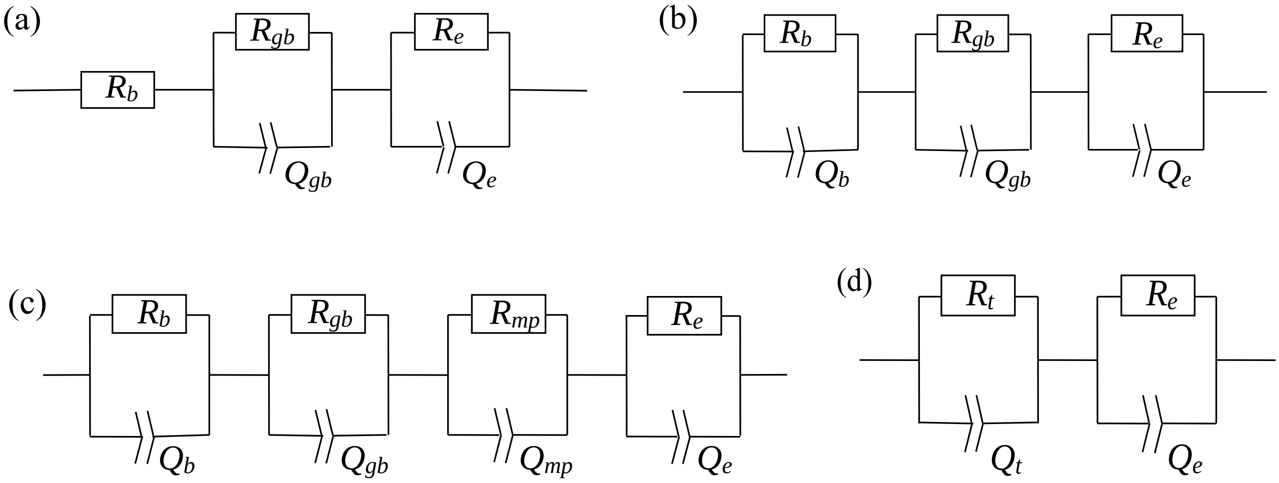

Figure S2. Equivalent circuits used for deconvolution of impedance spectra for: (**a**) x = 0.98 and 1.00 at 550–800 °C; (**b**) x = 0.98 and 1.00 at 500 °C and below; (**c**) x = 0.96 at 350 °C and below; (**d**) x = 0.94. R_t denotes the total resistance of a sample; R_b, R_{gb}, R_{mp} and R_e are the grain interior, grain boundary, minor phase and electrode resistances, respectively; Q_b, Q_{gb}, Q_{mp} and Q_e are the constant phase element associated with the grain interior, grain boundary, minor phase and electrode responses; Q_t is the constant phase element related with the total response of a sample.

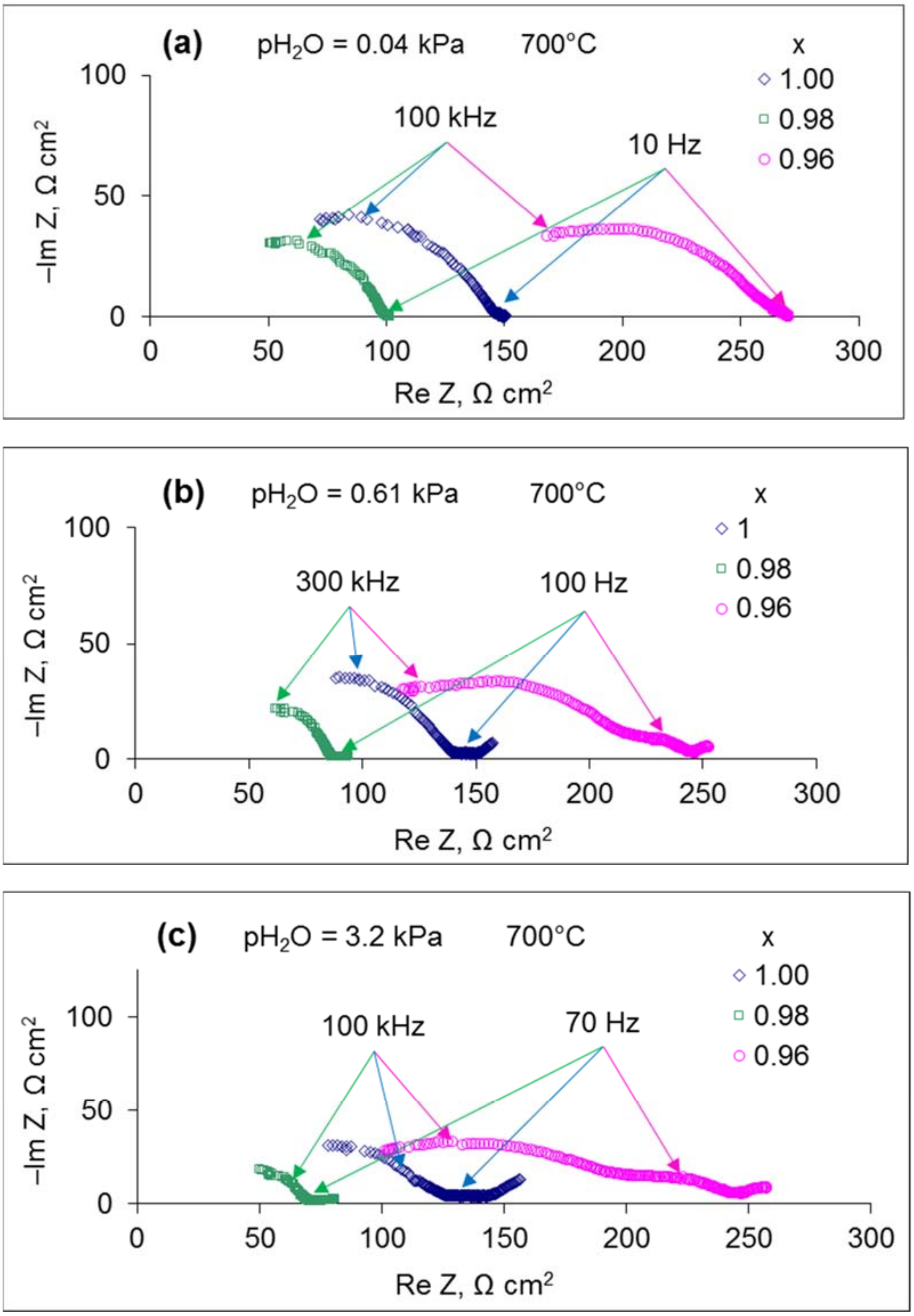

(a)
pH2O = 0.04 kPa
700°C
x
1.00
0.98
0.96
100 kHz
10 Hz
−Im Z, Ω cm2
Re Z, Ω cm2
0
50
100
150
200
250
300
(b)
pH2O = 0.61 kPa
700°C
x
1
0.98
0.96
300 kHz
100 Hz
−Im Z, Ω cm2
Re Z, Ω cm2
(c)
pH2O = 3.2 kPa
700°C
x
1.00
0.98
0.96
100 kHz
70 Hz
−Im Z, Ω cm2
Re Z, Ω cm2

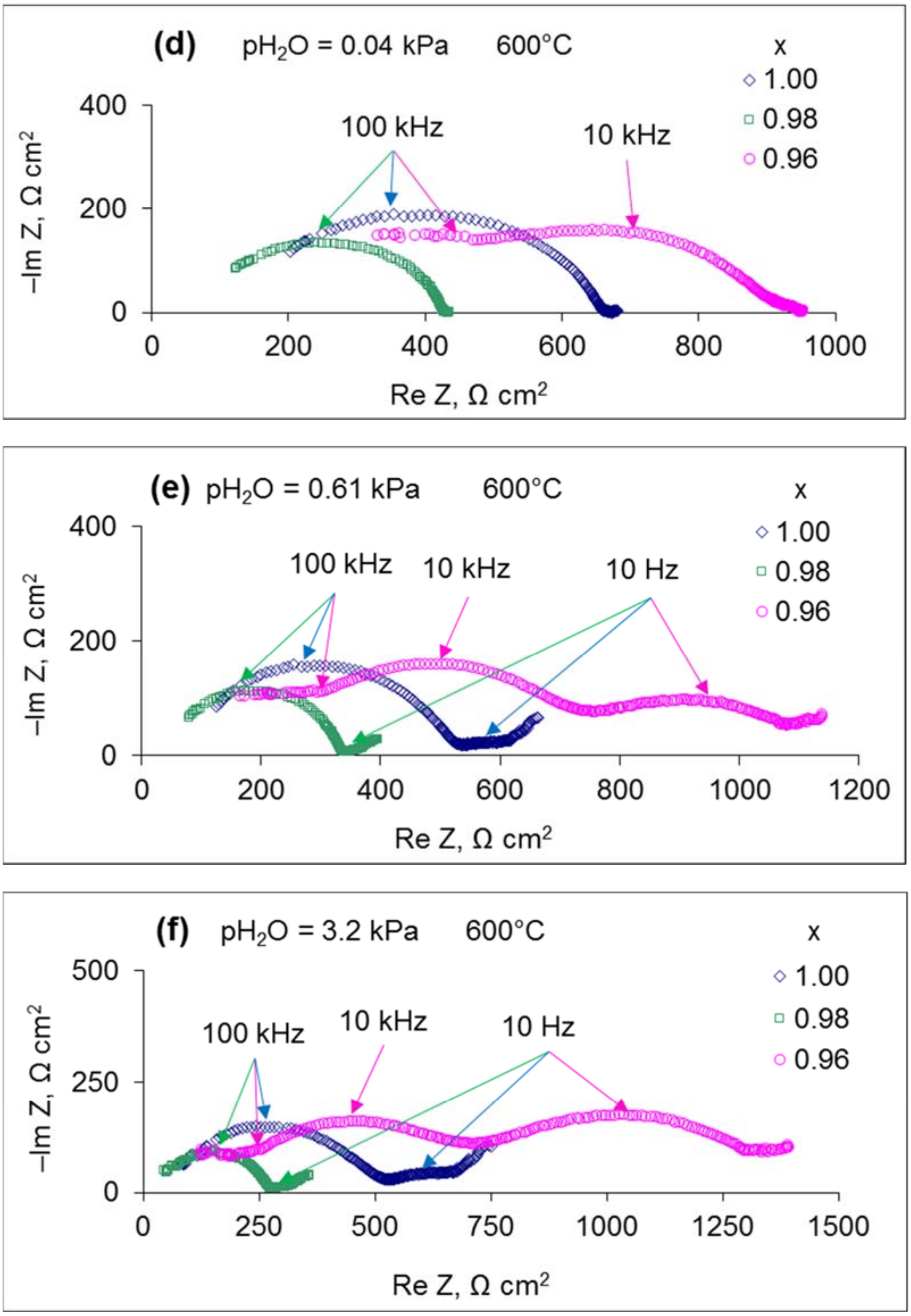
(d) pH2O = 0.04 kPa 600°C
x
1.00
0.98
0.96
100 kHz
10 kHz
−Im Z, Ω cm2
Re Z, Ω cm2
(e) pH2O = 0.61 kPa 600°C
100 kHz
10 kHz
10 Hz
(f) pH2O = 3.2 kPa 600°C
100 kHz
10 kHz
10 Hz

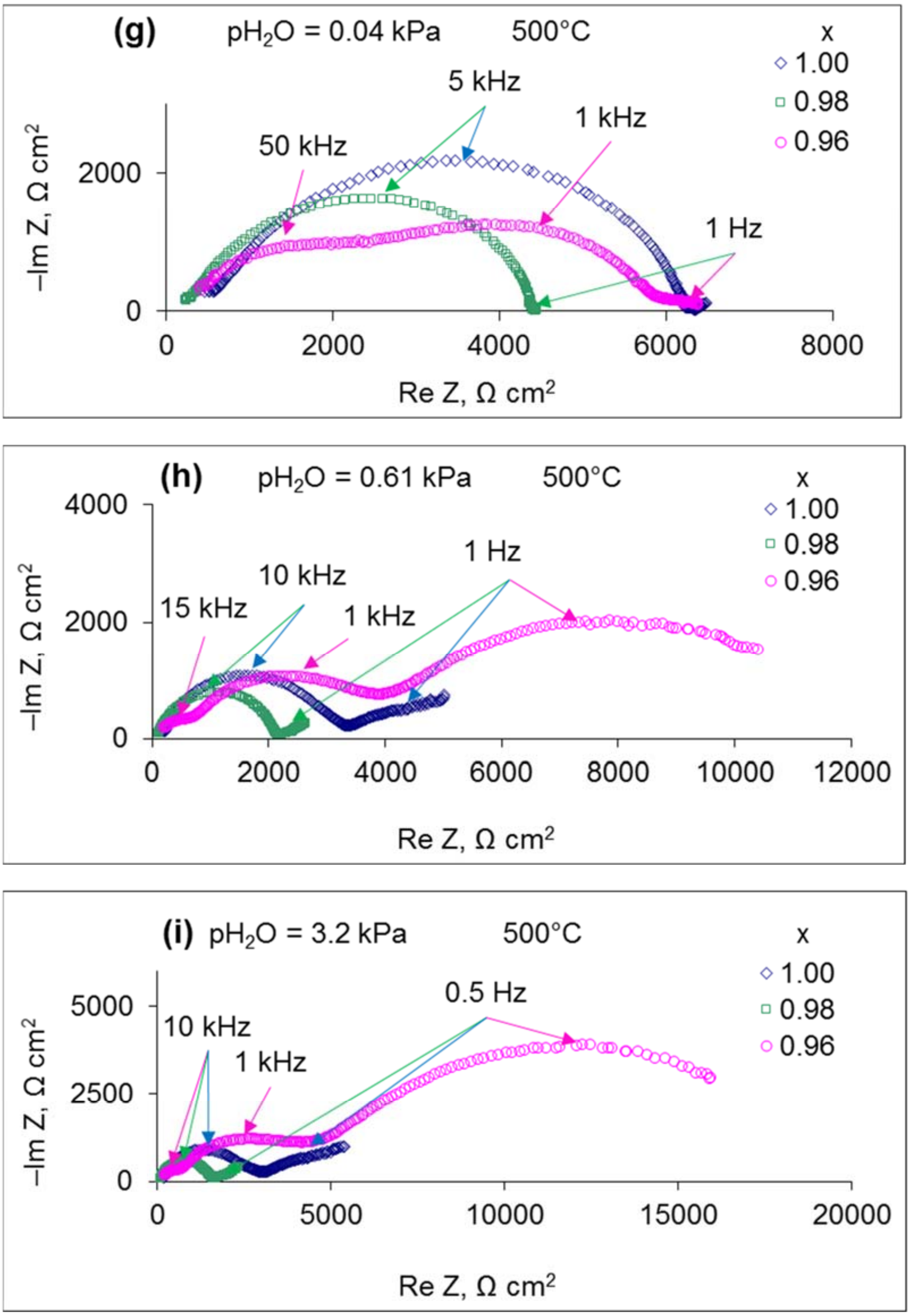
(g)
pH2O = 0.04 kPa
500°C
x
1.00
0.98
0.96
5 kHz
1 kHz
50 kHz
1 Hz
−Im Z, Ω cm2
Re Z, Ω cm2
(h)
pH2O = 0.61 kPa
500°C
x
1.00
0.98
0.96
1 Hz
10 kHz
15 kHz
1 kHz
−Im Z, Ω cm2
Re Z, Ω cm2
(i)
pH2O = 3.2 kPa
500°C
x
1.00
0.98
0.96
0.5 Hz
10 kHz
1 kHz
−Im Z, Ω cm2
Re Z, Ω cm2

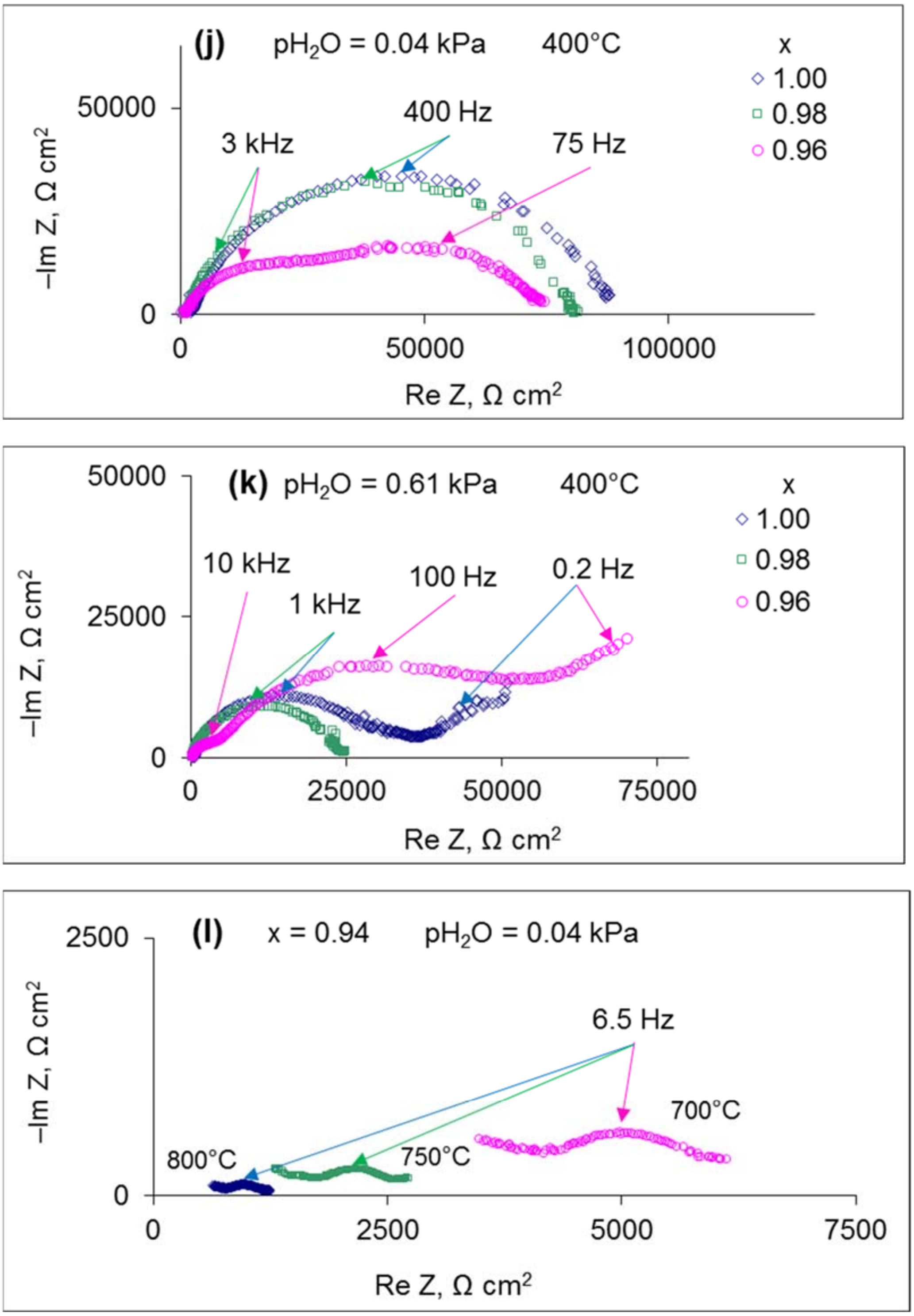

Figure S3. Impedance spectra of the samples with x = 0.96–1.00 measured in air at 700 °C (a–c), 600 °C (d–f), 500 °C (g–i) and 400 °C (j,k); at pH_2O = 0.04 kPa (a,d,g,j), 0.61 kPa (b,e,h,k) and 3.2 kPa (c,f,i); and the spectra of the x = 0.94 sample at pH_2O = 0.04 kPa (l).
